# A computer vision framework for quantification of feather growth patterns

**DOI:** 10.3389/fbinf.2023.1073918

**Published:** 2023-02-03

**Authors:** Tyler N. Thompson, Anna Vickrey, Michael D. Shapiro, Edward Hsu

**Affiliations:** ^1^ Department of BioMedical Engineering, University of Utah, Salt LakeCity, UT, United States; ^2^ University of Utah School of Biological Sciences, Salt LakeCity, UT, United States

**Keywords:** machine learning, clustering methods, feature extraction, bioimaging, point cloud processing

## Abstract

Feather growth patterns are important anatomical phenotypes for investigating the underlying genomic regulation of skin and epidermal appendage development. However, characterization of feather growth patterns previously relied on manual examination and visual inspection, which are both subjective and practically prohibitive for large sample sizes. Here, we report a new high-throughput technique to quantify the location and spatial extent of reversed feathers that comprise head crests in domestic pigeons. Phenotypic variation in pigeon feather growth patterns were rendered by computed tomography (CT) scans as point clouds. We then developed machine learning based, feature extraction techniques to isolate the feathers, and map the growth patterns on the skin in a quantitative, automated, and non-invasive way. Results from five test animals were in excellent agreement with “ground truth” results obtained *via* visual inspection, which demonstrates the viability of this method for quantification of feather growth patterns. Our findings underscore the potential and increasingly indispensable role of modern computer vision and machine learning techniques at the interface of organismal biology and genetics.

## 1 Introduction

**FIGURE 1:**
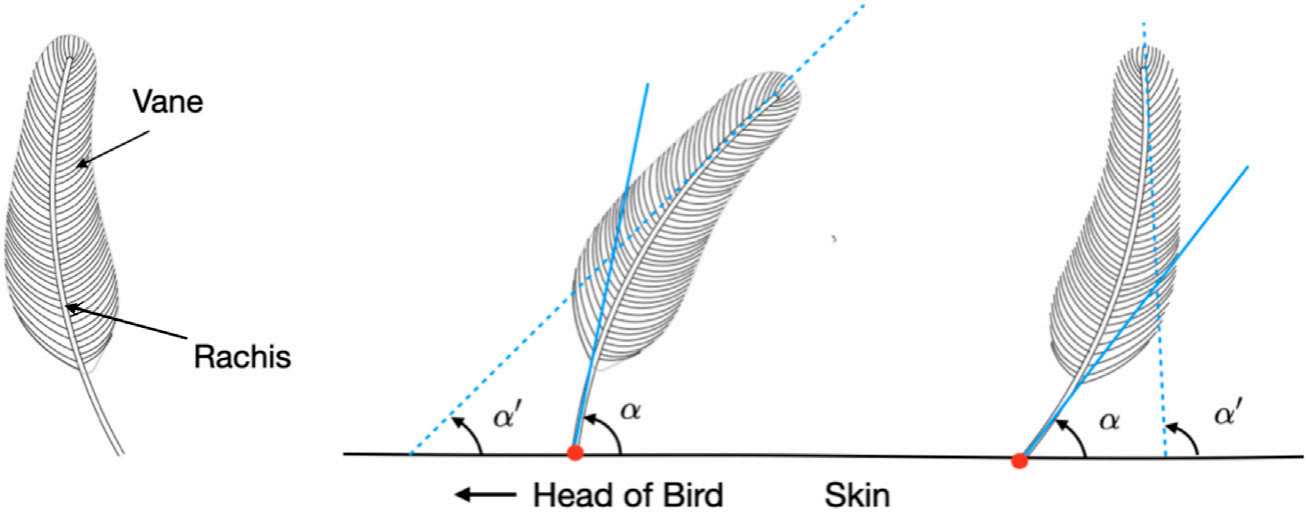
Schematics of single feathers. In general, a feather (left) consists of the rigid central shaft, called the rachis, which supports the softer fan-shaped vane, composed of interlocking barbs. The polarity of a feather can be mathematically modeled and determined from the angles *α* and *α*′ formed by the tangent lines at the tip and base of its rachis, respectively, and the plane of the skin surface. A feather can be categorized as having normal (center) or reverse polarity (right) based on whether Δ*α* = *α* − *α*′ is positive or negative.

**FIGURE 2:**
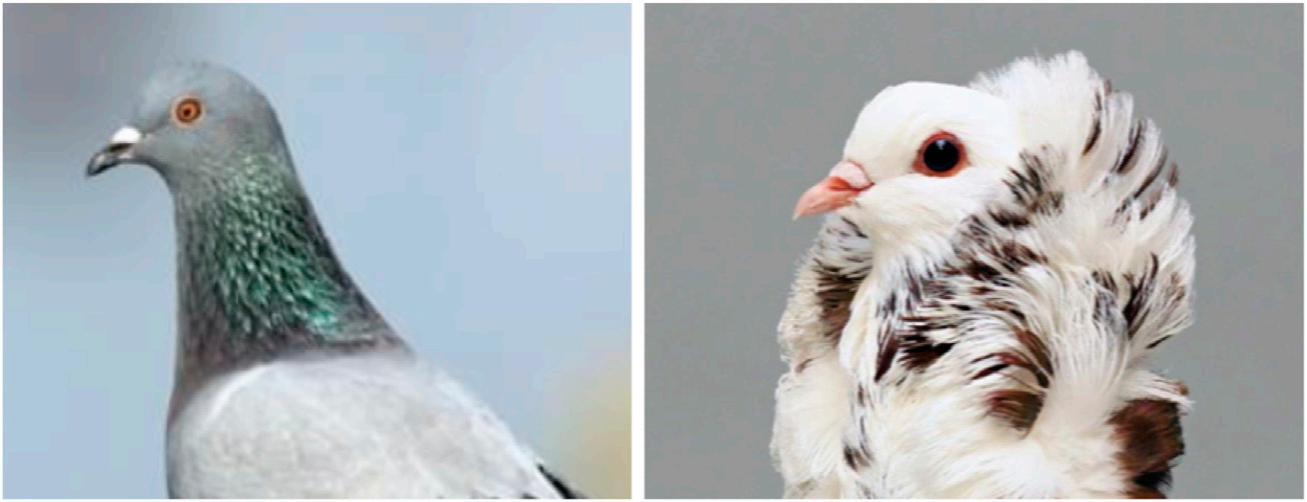
Head crest phenotypes of domestic rock pigeons. In contrast to a pigeon without head crest (left), a crested pigeon is characterized by a patch of feathers with reversed polarity on the skin of the head and neck (right). Credit: The Macaulay Library at the Cornell Lab of Ornithology (ML61674401), Sydney Stringham.

Variations in animal morphology often have a genetic origin. For example, many bird species exhibit diverse patterns of head crests, which are plumage display structures associated with communication and courtship (Prum, 1999; Amundsen, 2000; Price, 2002). In domestic rock pigeons (*Columba livia*), head crests are characterized by feathers on the back of the head and neck that are reversed in their growth polarity: the normal dorsal side becomes ventral and the ventral side is dorsal, resulting in a feather that curves up toward the top of the head instead of down and away from it (Figure 1). Head crests vary among pigeon breeds in both the size of the feathers and their spatial extent (Figure 2). A variant in the gene EphB2 appears to be necessary for head crest formation, but this gene alone does not explain all of the quantitative and qualitative variation in head crests throughout the species (Shapiro et al., 2013). Therefore, quantitative assessments of head crest morphologies are essential to discover additional genes that control crest variation. Identification of these genes, in turn, will broaden our understanding of the genetic and developmental mechanisms that regulate patterning and growth of skin appendages in vertebrates (*e.g.,* feathers, hair, scales).

Feathers have a long history in developmental biology as a model for growth, patterning, and regional skin identity (Boer et al., 2017). However, studies of adult feather patterns and morphology largely rely on tedious manual examination and visual inspection of individual feathers. Measuring large tracts or patches of feathers can be highly labor-intensive, time-consuming, and error-prone. These challenges led the authors of one study to conclude that, “The work of feather counting is tedious and exacting and yields small result relative to the labor involved” (Wetmore, 1936). The ideal approach for examining feather patterns should be based on non-invasive imaging, which allows morphological information to be captured *in situ*, without perturbing or removing the feathers. Moreover, the approach should be quantitative, so that the measurements can be readily integrated into data science analysis pipelines for genetic and genomic analyses to map the genes that control variation. For example, to understand head crest variation in rock pigeons, the most relevant measurements are the location, density, and extent of the skin of the head and neck where the feathers grow with reversed polarity. Genetic mapping projects typically require large numbers of animals for robust statistical support, (Hong and Park, 2012) so an analysis of feather patterning should also be sufficiently fast and fully-automatable.

Here, we present an approach for quantifying feather patterns that utilizes (a) non-invasive computed tomography (CT) imaging, (b) basic post-processing to convert the images into point cloud representations which capture the morphology of the skin and feathers, (c) cluster and machine learning analyses to isolate and identify individual feathers, and (d) numerical procedures to quantify feather curvature and extrapolate the location and area of reversed feathers. Our results, which are validated by visual inspection, demonstrate the feasibility and utility of combining imaging and machine vision to achieve what was once thought to be a practically impossible analysis. We anticipate our approach will have broad utility in various fields beyond avian biology.

## 2 Materials and methods

### 2.1 Image acquisition

CT captures the density of tissues and materials, and has been used on birds (Jones et al., 2019; Boer et al., 2021), though the scans were optimized mostly for internal hard-tissue anatomy. Visualization of feathers was challenging due to their low radiopacity. For the current study, freshly sacrificed domestic rock pigeons (*n* = 8) were carefully handled to preserve the natural conformation of the head and neck with minimal perturbation to the feathers, and scanned at 50 *μm* resolution using a small-animal CT instrument (Inveon, Siemens Preclinical Imaging). The acquisition settings, which included relatively low x-ray energy of 30 kV but high current of 500*μA*, were empirically determined to provide maximum contrast between the feathers and surrounding air. The scans were then reconstructed using a standard Feldkamp algorithm with Shepp-Logan filtering (Gullberg, 1979), and converted to Houndsfield units (HU).

### 2.2 Anatomical representation

All post-processing and analysis were conducted on a Linux workstation equipped with an AMD Ryzen 1900X 4.0 GHZ CPU, 64 GB of RAM, and an NVIDIA GTX 2080-TI graphics card. As the first step in the post-analysis, the anatomical features in the CT scans needed to be represented mathematically (*e.g.,* converting into 3D coordinate points) to facilitate their quantitative characterization. While this could be accomplished *via* several different methods, we rendered the CT volume as isosurfaces using Amira with empirically-determined intensity ranges, binarizing the data within the intensity window. We then generated point clouds from the vertices of the polygons that made up the isosurfaces. The coordinates of the surface element vertices obtained using a high intensity threshold (−200 HU) were grouped directly into the point cloud denoting “skin”. In contrast, the “everything” point cloud was generated using a low threshold (−860 HU) to remove background noise. Subsequently, “skin” was excluded from “everything” to form the “feathers” point cloud, by removing points within the “everything” cloud in close proximity to vertices in the “skin” cloud. The remaining “feathers” cloud contains mostly feather rachises, and residual image noise and artifacts.

### 2.3 Cluster isolation and identification

Before quantifying the morphological features, another necessary step was to identify and isolate the point clusters associated with individual feathers in the “feathers” point cloud, which contained not only the rachises, but also imaging artifacts and unremoved image noise. Fortunately, akin to visual inspection based on anatomical contiguity, points of an individual feather could be isolated by the proximity or density of its points. To this end, a python implementation of the Hierarchical Density-Based Spatial Clustering of Applications with Noise (HDBSCAN) algorithm (Campello et al., 2013; McInnes et al., 2017) was used to identify the clusters of points associated with each distinct object. The clustering algorithm was applied to the aforementioned “feather” point cloud, the default algorithm parameters in the referenced implementation were used with the exception of minimum allowable cluster size which was set to 50 points. As point clouds are intrinsically unordered, the identified clusters were indexed and ordered with respect to distance from the “skin” point cloud. Each indexed cluster was then condensed to 10 equally spaced points by averaging spatial locations along the indexed dimension. This process is demonstrated in Figure 5. To classify each cluster as either “feather” or “not feather”, a single channel convolutional neural network consisting of three convolutional layers followed by a six layer classification network with leaky ReLU activation functions was implemented in Pytorch (Adam et al., 2019). Each of the subsampled clusters was represented as a 10 by three array consisting of the normalized xyz coordinates of each of the 10 points. To generate training data for the model, three birds (one crested, and two un-crested) were scanned and processed to generate point clouds. This process took about 18 h, and generated approximately 30,000 clusters, of which 22,000 were used to train the model, and the remaining 8,000 were used for testing. At training time, the model was trained for 120 epochs, updated in accordance with the default Pytorch implementation of the ADAM optimization algorithm, and evaluated on the test clusters, taking just under 2 hours.

### 2.4 Morphological quantification

Following isolation and identification of the point clusters associated with individual feathers, the desired morphological quantification could be obtained numerically. The polarity of each of the identified feathers was obtained by calculating linear approximations to both the top and bottom of the feather as demonstrated in Figure 1., using the condensed points as demonstrated in Figure 5. The two linear approximations are calculated using the top three and bottom three points of the condensed points, and are used to extrapolate intersection points from which the angles *α* and *α*′ can be determined. These intersection angles are then compared to each other to generate the Δ*α* term. Subsequently, the feathers were labelled as having “normal” or “inverted” polarity, depending on whether Δ*α* is positive or negative, respectively. Furthermore, the extrapolation of the bottom feather points were used to determine the insertion points of the feathers on the skin surface. The insertion points of feathers with inverted polarity were used to calculate the local crest feather density on the skin surface coordinate. Finally, the skin region where feather density was greater than the empirically determined threshold of 7 $\frac{\text{feathers}}{{\text{cm}}^{2}}$ was taken to be the head crest area.

### 2.5 Performance assessments

The performance of our feather pattern analysis framework was evaluated in two ways. The accuracy of the cluster classification was evaluated on the 8,000 clusters in the test set which were hand labeled. Second, the final outputs of the analysis pipeline (the areas of the skin occupied by the inverted polarity growth pattern) was evaluated on 5 birds separate from the ones from which the training clusters were derived. To perform the evaluation, the clusters from the “feather” point cloud for each bird were manually labeled (taking about 6 h per bird to hand label each of the clusters), and calculated polarity verified. With the manually verified quantities, the remainder of the process is carried out to map the region of inverted polarity. The intersection-over-union is then calculated between this area and the area determined by the fully automated process. As a control, the same procedure was done on two additional birds without head crests to verify that no area was identified.

## 3 Results

**FIGURE 3:**
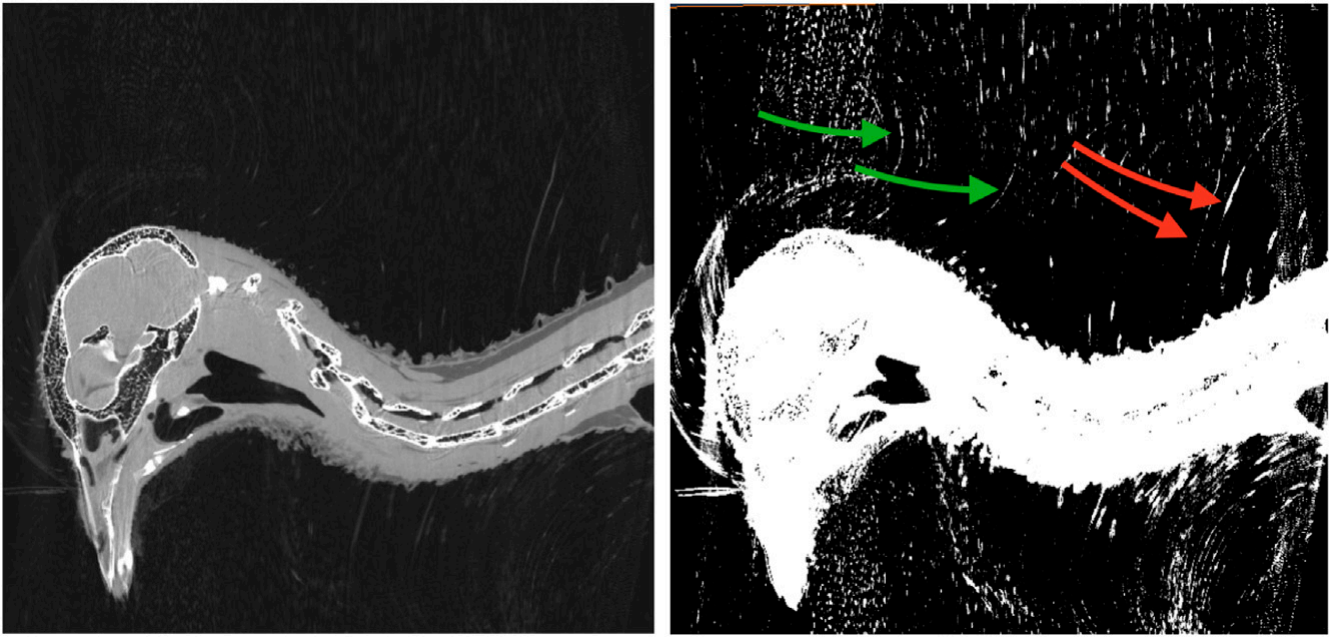
CT scan of a rock pigeon head. A single sagittal slice is shown in both normal (left) and saturated (right) intensity scales. The shafts of the inverted feathers (green arrows), and conventional feathers (red arrows) are visually discernible, but difficult to distinguish from the background noise and artifacts.

**FIGURE 4:**
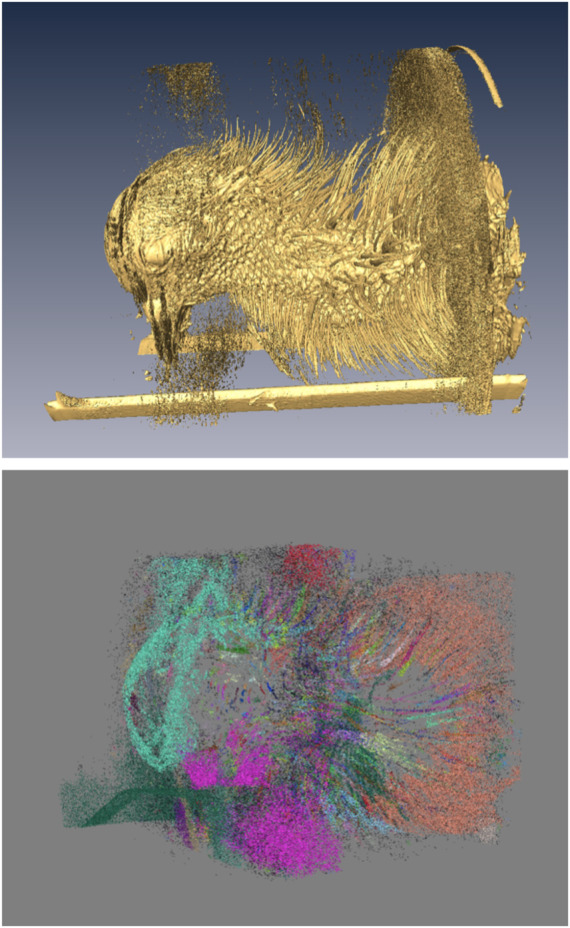
Generation of morphological point clusters. A low-intensity threshold was applied to obtain the “everything” isosurface (top), whose vertices were used to form the corresponding point cloud. A “skin” point cloud (not shown) was similarly obtained using a high-intensity threshold. The “feathers” point cloud (bottom) was produced by excluding the “skin” from the “everything” cloud. Clusters corresponding to individual objects identified from the point cloud are shown in different colors. In the isosurface rendering (top), the rectangular structures at the bottom are parts of the scanner animal holding bed, and the patches of dispersed small objects above and below the head, and at the base of the neck, are imaging noise and artifacts. Note that the isosurface on top is generated at a slightly higher intensity to remove some of the noise present in the below point cloud to better display the feathers which are being identified. In the implementation of the pipeline, the points in the isosurface and the point cloud match exactly.

**FIGURE 5:**
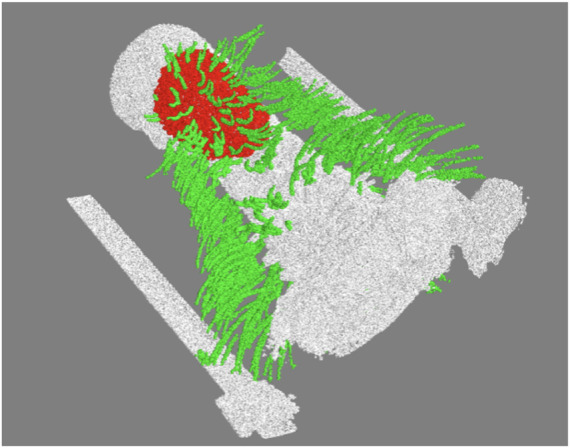
Example of clusters classified as feathers (green), the skin point cloud (white), and the region of inverted polarity (red).

**FIGURE 6:**
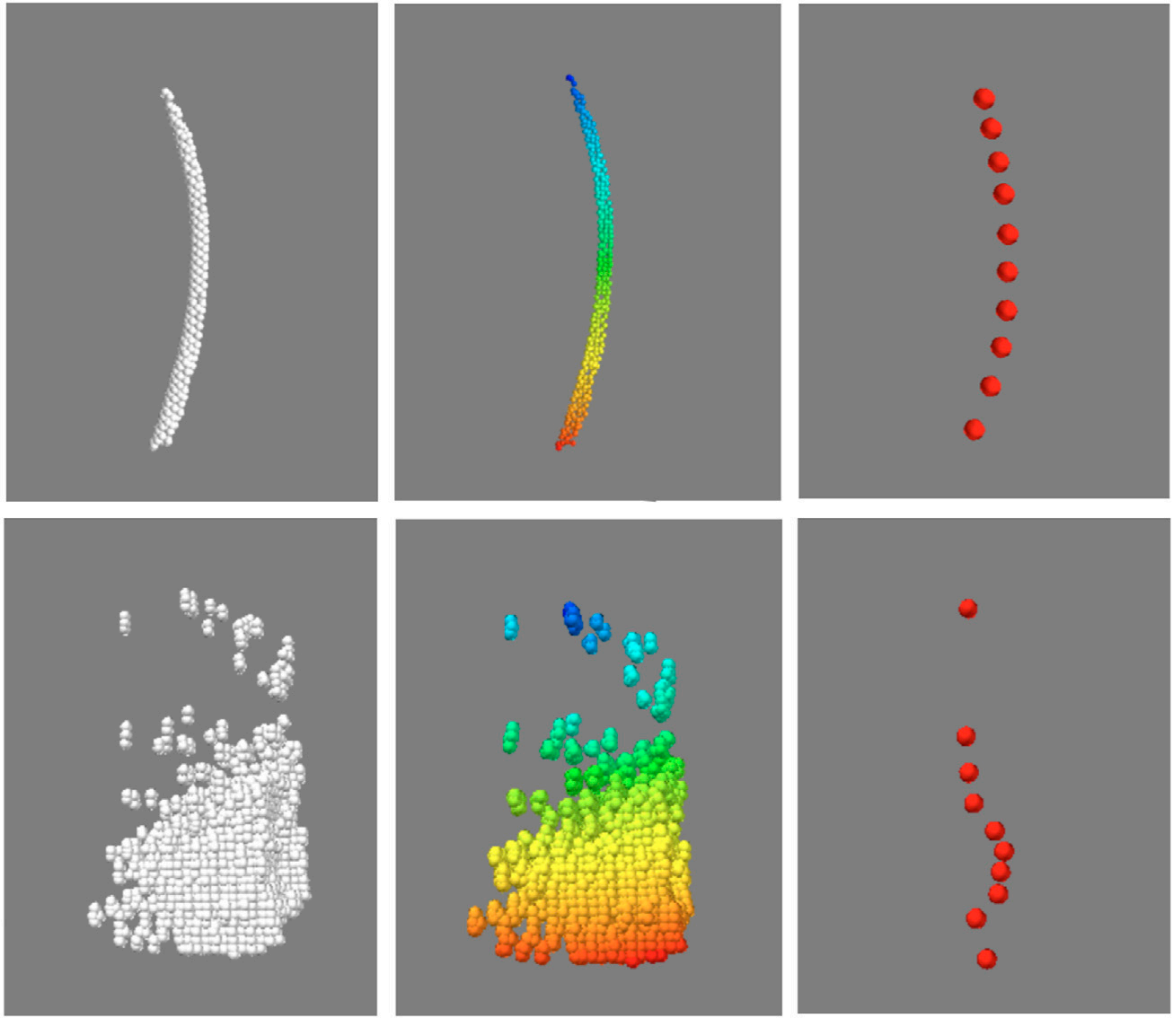
Sorting and sub-sampling of individual clusters for feather identification. The top and bottom panels correspond to representative feather and non-feather clusters. The unsorted clusters (left) are sorted by distance with respect to one end of the feather, and falsecolor coded for visualization, then sub-sampled (right). Despite of the reduction in the number of points in each object, essential hallmarks of the feather are still recognizable.

**FIGURE 7:**
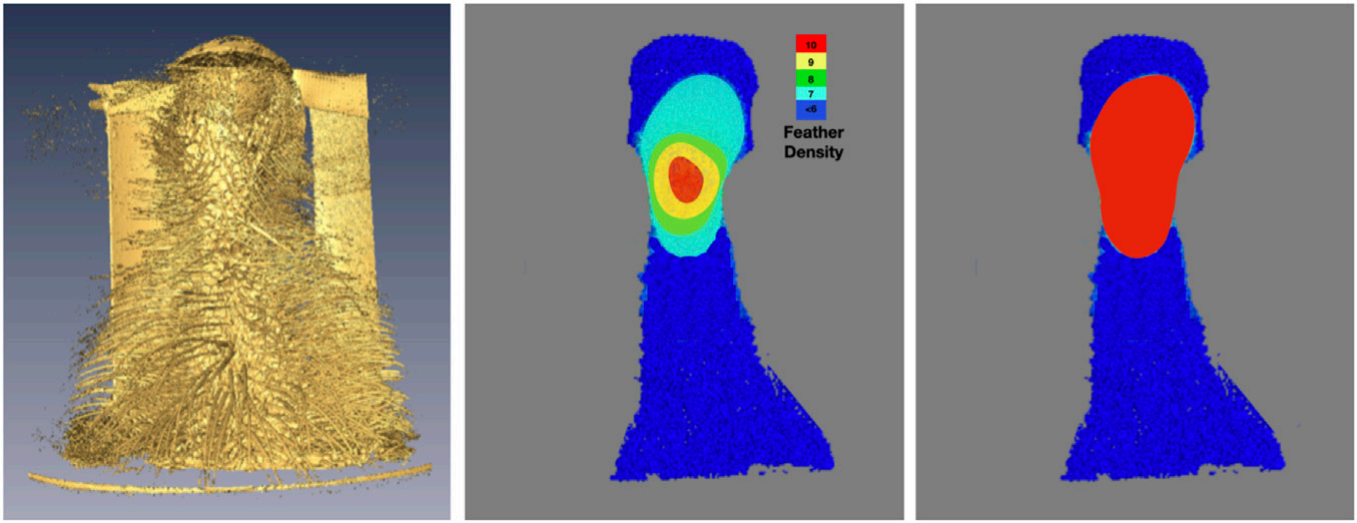
Determination and visualization of the crest. Using the isosurface rendering (left) of the skin and feather shafts as reference, the density of feathers with inverted curvature is shown in falsecolor (center). The crest, defined here as region where inverted-feather density is greater than seven feathers per square centimeter, is obtained *via* simple thresholding the density and shown as a binary map (right).

**FIGURE 8:**
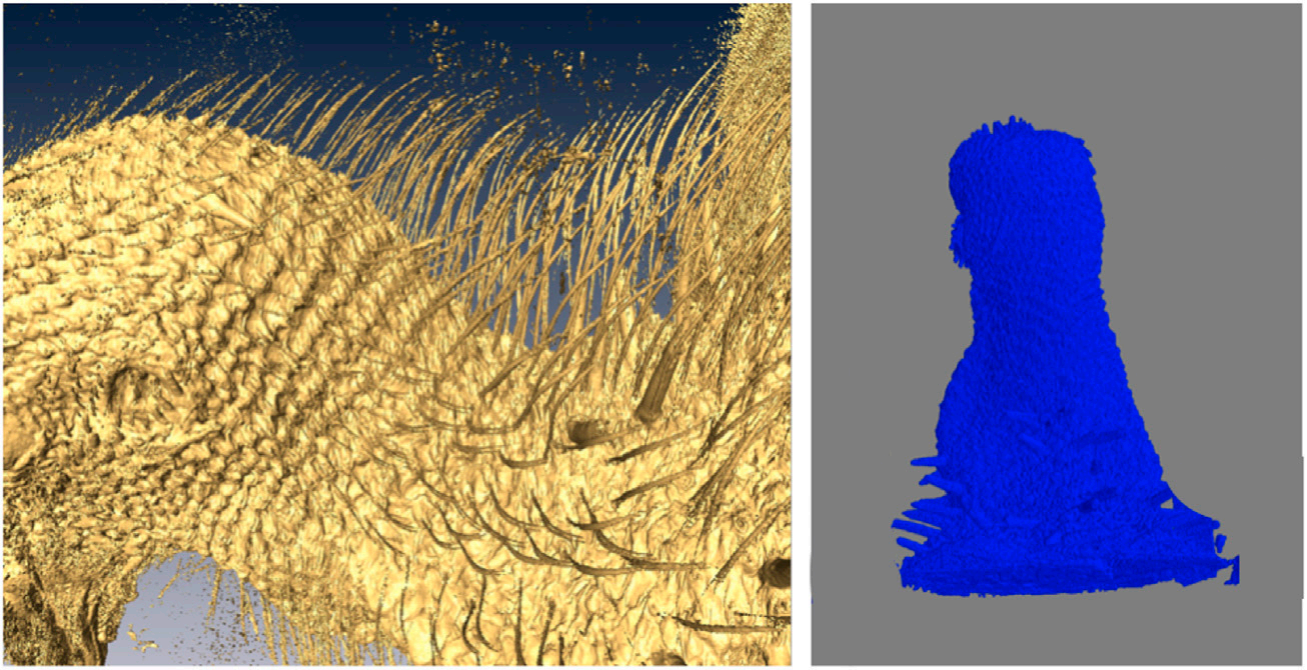
A representative bird without crest used as control. Close-up of the CT isosurface rendering reveals feathers that all have normal polarity (left). The automated image analysis pipeline did not detect any crest area (right).

A representative CT image of a pigeon head and neck is shown in Figure 3. As expected, the image captured exquisite details of the interior anatomy (e.g., bones). In contrast, the shafts of the feathers, background noise and artifacts have similarly low intensity, which underscores the difficult but not impossible tasks of the subsequent analyses. Figure 4 shows an anatomical isourface and a point cloud used for cluster and morphological analyses, these clusters are classified as being a feather or not, the clusters identified as feathers are displayed in Figure 5 in green, while the skin is denoted in white, and the region determined to have inverted polarity in red. Figure 6 shows a representative feather and non-feather point clusters at the three stages of pre-classification processing, detection, ordering, and subsampling respectively from left to right. The final outputs of the current analysis pipeline, which are the density map of inverted-polarity feathers and binary region denoting the crest location and extent, are shown in Figures 7, 8 for a crested and non-crested bird, respectively.

**FIGURE 9:**
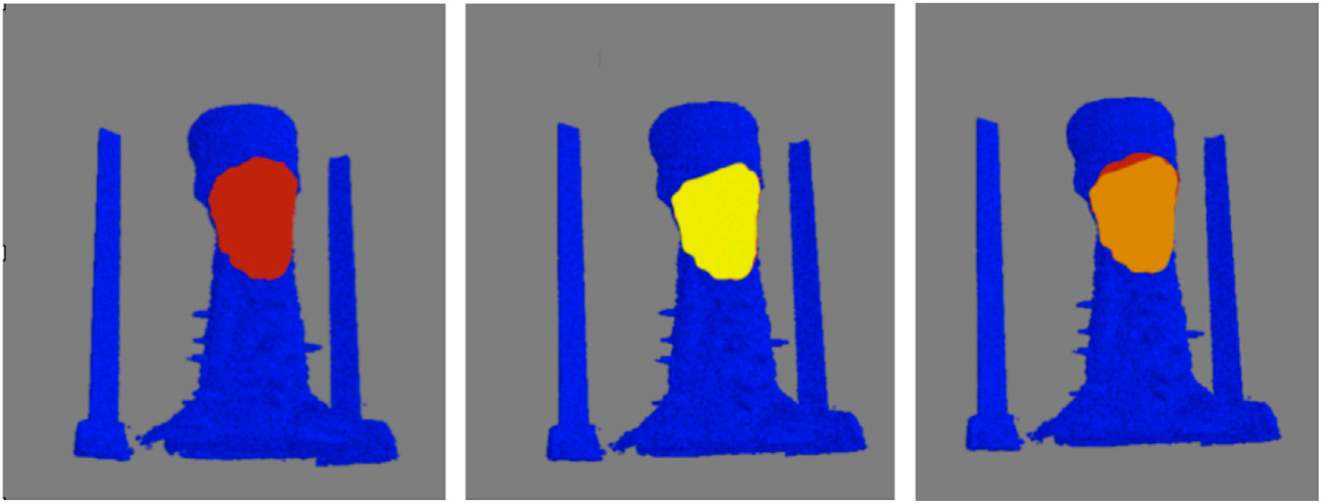
Verification of determined crest area for a representative bird. Top-view of the crest areas determined by computer (left) and visual inspection (center) are shown in maroon and yellow, respectively, on blue-color surface rendering of the skin. The overlap of the areas is shown in orange (right). For this particular animal, the computer-determined crest area is slightly smaller (4%) than that by visual inspection.

As metrics of performance, among the 8000 individual clusters compared, the automated (i.e., machine learning) classification correctly identified the feather clusters 86.0% of the time, while producing 4.5% and 9.4% false-positive and false-negative identifications, respectively. Although the accuracy could likely be improved by additional training data, more sophisticated algorithms, and additional training, the performance was deemed acceptable for characterizing the collective behavior of regions of feathers (*e.g.,* density and area of reversed feathers) rather than the individual feather polarity. For the second test of performance, the intersection over union was calculated for 5 birds, three with crests, and two without. Figure 9 shows a representative comparison between the crest area obtained *via* computer and visual inspection. Among the three crested birds examined, the automated and visually-determined crest areas overlap by an average of 95.0% ± 3.1% (mean ± standard error of the mean). In contrast, no crest area was detected for either of the two animals without a crest, as expected. Together, these findings indicate that our proposed framework for feather characterization is highly accurate and reliable.

In terms of computational performance, the entire analysis (from point cloud creation to mapping of the growth pattern) took approximately 4.8 min per bird on our modest computation setup. Beyond initial empirical determination of the computation parameters (e.g., intensity threshold levels), the analysis was done in a fully automated fashion without additional user input.

## 4 Discussion

We applied computer vision and machine learning techniques to analyze feather morphologies from CT images of pigeons, and quantified the location and area of their head crests. The results are in excellent agreement with the current “gold standard” obtained by visual inspection. It is worth re-iterating that while this approach was only applied to one species of bird, we see no reason why the technique should not be generalizable to different species which also exhibit the head crest growth pattern, and could be adapted to quantify different types of growth patterns.

Excluding initial empirical determination of the parameters, and labelling of the training data for the cluster identification model, the entire analysis was performed automatically and took less than 5 min processing time on our modest workstation. Compared to the CT scan and reconstruction times of 57 and 23 min, respectively, our analysis will not become a bottleneck in a high-volume and high-throughput project. To our knowledge, this is the first time that the analysis of feather growth patterns is successfully performed in a non-invasive, quantitative and fully-automated fashion.

In order for this approach to be viable, the structures being mapped must satisfy two primary criteria. They have to exist within a radio-opacity threshold so that they can be rendered as an isosurface, and be separable into individual structures *via* clustering or some similar method. Beyond elucidating quantifiable metrics in feathers, it is possible that there is applicability of this technique to other topologically analogous scenarios which satisfy the criteria. Limiting the discussion to biological scenarios, there are many examples of these so-called “over-growth” structures where different tissue types grow directly on top of on another. For example, osteosarcomas are cancerous tumors which grow on the surface of bones (Widhe and Widhe, 2000; Roller et al., 2018). Similarly, Paget’s disease is characterized by cartilaginous and fibrous outgrowth structures on the spine (Saifuddin and Hassan, 2003). The technique we’ve developed for quantifying the morphology of feather growing out of the skin may be a viable approach for identifying and quantifying the morphology of these types of biological overgrowth structures characterized by one tissue type growing immediately next to another.

Although the results are encouraging, the current study has several potential limitations. First, the results are validated by comparing to those obtained *via* visual inspection. Although it currently produces the best-available “ground truths”, visual inspection is necessarily subjective, and its labor-intensiveness limits the number of comparisons that can be practically performed. Second, the accuracy of our machine learning algorithm for identifying feathers is a modest 86%, and the accuracy in determining their polarity has not been directly evaluated. Although our current outputs (i.e., the overall location and area of the head crest) appear not to have been adversely impacted, these limitations may need to be addressed in other applications. One notable approach could be *via* additional training data (more hand labeled clusters) or data augmentation. In early versions of the machine learning based cluster classification, the use of data augmentation was explored, but in order to make the approach robust to relative position of birds in the scanner, location of feathers, and computational stability, the xyz points used for classification were normalized. This normalization made the most straightforward methods of data augmentation like rotation, translation, dilation of clusters less representative of the actual data. In future work, or where higher accuracy on the cluster identification task was necessary, more complex data augmentation methods could certainly be explored.

Lastly, as a pipeline for image analysis, the performance is highly dependent on the modality used and quality of the input images. The current study was possible only after the optimal CT protocol for maximizing the contrast of the feather rachises in the CT images was determined. The imaging and analysis pipeline likely needs to be optimized on a case-by-case basis for each different application.

## Data Availability

The raw data supporting the conclusion of this article will be made available by the authors, without undue reservation.
